# A Novel Sweetpotato WRKY Transcription Factor, IbWRKY2, Positively Regulates Drought and Salt Tolerance in Transgenic *Arabidopsis*

**DOI:** 10.3390/biom10040506

**Published:** 2020-03-27

**Authors:** Hong Zhu, Yuanyuan Zhou, Hong Zhai, Shaozhen He, Ning Zhao, Qingchang Liu

**Affiliations:** 1 Key Laboratory of Sweetpotato Biology and Biotechnology, Ministry of Agriculture and Rural Affairs/Beijing Key Laboratory of Crop Genetic Improvement/Laboratory of Crop Heterosis and Utilization, Ministry of Education, College of Agronomy & Biotechnology, China Agricultural University, Beijing 100193, China; zhuhong198812@126.com (H.Z.); yy_zhou@cau.edu.cn (Y.Z.); zhaihong@cau.edu.cn (H.Z.); sunnynba@cau.edu.cn (S.H.); zhaoning2012@cau.edu.cn (N.Z.); 2 College of Agronomy, Qingdao Agricultural University, Qingdao 266109, China

**Keywords:** sweetpotato, *IbWRKY2*, *Arabidopsis*, drought and salt tolerance, VQ4

## Abstract

WRKYs play important roles in plant growth, defense regulation, and stress response. However, the mechanisms through which WRKYs are involved in drought and salt tolerance have been rarely characterized in sweetpotato [*Ipomoea batatas* (L.) Lam.]. In this study, we cloned a *WRKY* gene, *IbWRKY2*, from sweetpotato and its expression was induced with PEG6000, NaCl, and abscisic acid (ABA). The IbWRKY2 was localized in the nucleus. The full-length protein exhibited transactivation activity, and its active domain was located in the N-terminal region. *IbWRKY2*-overexpressing *Arabidopsis* showed enhanced drought and salt tolerance. After drought and salt treatments, the contents of ABA and proline as well as the activity of superoxide dismutase (SOD) were higher in transgenic plants, while the malondialdehyde (MDA) and H_2_O_2_ contents were lower. In addition, several genes related to the ABA signaling pathway, proline biosynthesis, and the reactive oxygen species (ROS)-scavenging system, were significantly up-regulated in transgenic lines. These results demonstrate that *IbWRKY2* confers drought and salt tolerance in *Arabidopsis*. Furthermore, IbWRKY2 was able to interact with IbVQ4, and the expression of *IbVQ4* was induced by drought and salt treatments. These results provide clues regarding the mechanism by which *IbWRKY2* contributes to the regulation of abiotic stress tolerance.

## 1. Introduction

Ever-changing environmental stresses, such as drought, high salinity, and extreme temperature, have become increasingly major constraints for crop production [1,2]. Drought and soil salinity are two major devastating problems in agriculture and are becoming particularly widespread; therefore, the need to raise the levels of drought and salt tolerance in crops has become crucial [3,4,5]. To respond and adapt to these environmental stresses, plants activate a series of elaborate and sensitive defense mechanisms to promote their survival [5,6]. The mechanisms of stress tolerance are complex because they can be affected by not only the severity and duration of the stress event but also the plant developmental stage and morphology [7,8]. In plants, a change in the expression of genes, especially transcription factors (TFs), is generally the earliest response to stress conditions, and TFs often act as central regulators and molecular switches in stress signal transduction and adaptation networks [9,10,11]. A single TF can control the expression of several target genes by binding to a specific element present in their promoters [5]. To date, a large number of TF families, such as NAC, MYB, bHLH, and WRKY, have been demonstrated to participate in the regulation of stress responses and tolerance in plants [12].

Among the different TFs, the WRKY family, originally isolated from sweetpotato, is one of the largest families and has received increasing attention for its roles in plant defense [13,14]. The members of the WRKY TF family possess at least one conserved 60 aa WRKY domain containing a highly conserved WRKYGOK amino acid sequence motif [13]. According to the number of WRKY domains and the type of zinc finger motif, the WRKY family can be divided into three distinct groups [15]. Members containing two WRKY domains belong to group I, whereas only one WRKY domain exists in group II and III members, which are distinguished by their zinc finger motif [15]. The functions of several WRKY TFs have been demonstrated in many kinds of plants using genetic and molecular approaches. WRKY members can form complex regulatory networks that are involved in plant responses to various stresses as well as many developmental processes [16,17]. In *Arabidopsis*, several WRKY TFs, such as AtWRKY8, AtWRKY28, AtWRKY54, and AtWRKY70, have been shown to mediate abiotic stress tolerance [18,19,20]. *OsWRKY11* and *OsWRKY30* overexpression lines showed dramatically increased drought tolerance in rice [21,22]. Overexpression of *TaWRKY2* and *TaWRKY19* displayed enhanced salt and drought tolerance in *Arabidopsis* [23]. Recent studies have shown that several WRKY proteins physically interact with VQ proteins to mediate various physiological processes [24]. VQ proteins are named for their conserved VQ motif, and several members have been demonstrated to play crucial roles in plant development and stress responses [24,25,26,27].

Sweetpotato, *Ipomoea batatas* (L.) Lam., is a globally important food crop because of its numerous advantages, such as strong adaptability, abundant nutrient content, stability, high yield, low input requirements, and diverse uses [28]. Its productivity is often limited by drought and salinity stresses, but the mechanism of resistance to these stresses remains unclear. To date, few of the WRKY TF family members have been functionally verified in sweetpotato. In this study, a novel WRKY transcription factor gene named *IbWRKY2*, belonging to the group I WRKY TF family, was isolated from the drought-tolerant sweetpotato line ‘Xushu55-2’. Overexpression of this gene enhanced drought and salt tolerance in transgenic *Arabidopsis*. IbWRKY2 was able to interact with both IbVQ4 and AtVQ4. The expression level of *IbVQ4* was induced by drought and salt treatments in sweetpotato. These results suggest that IbWRKY2, through its interaction with VQ4, may act as a positive regulator of abiotic stress tolerance, which provides key clues that further the current understanding of its roles in sweetpotato.

## 2. Materials and Methods

### 2.1. Plant Materials

The drought-tolerant sweetpotato line ‘Xushu55-2’ was employed to isolate the *IbWRKY2* gene. The expression profile of *IbWRKY2* was detected in ‘Xushu55-2’ treated with multiple abiotic stresses in this study. The plants were cultured on Murashige and Skoog (MS) medium for 4 weeks at 27 ± 1 °C under 13 h of daylight at 54 µmol m^−2^s^−1^. *Arabidopsis thaliana* (Columbia-0, WT) grown in a greenhouse (22 °C, 16/8 h day/night cycle) was used to characterize the function of *IbWRKY2*.

### 2.2. Cloning and Sequence Analysis of IbWRKY2 and Its Promoter

The total RNA of ‘Xushu55-2’ was extracted using the RNAprep Pure Plant Kit (Tiangen Biotech, Beijing, China), and first-strand cDNA synthesis was performed using PrimeScript™ II 1st Strand cDNA Synthesis Kit (TaKaRa, Beijing, China). According to the expressed sequence tag (EST) obtained from the drought transcriptome data of ‘Xushu55-2’, the rapid amplification of cDNA ends (RACE) method was used to obtain the full-length cDNA of *IbWRKY2* with the specific primers *IbWRKY2*-5’RACE-OUTER/INNER (Table S1) [29]. The sequence of *IbWRKY2* cDNA was analyzed by the NCBI (https://blast.ncbi.nlm.nih.gov/Blast.cgi). The open reading frame (ORF) of *IbWRKY2* was predicted using ORF Finder (https://www.ncbi.nlm.nih.gov/orffinder/). Multiple protein sequence alignments of WRKY2 were conducted with the DNAMAN software (Lynnon Biosoft, Quebec, Canada). The molecular weight and theoretical isoelectric point (pI) of IbWRKY2 were determined using ExPASy (http://web.expasy.org/compute_pi/). A phylogenic tree was constructed using MEGA 10.0 software with the neighbor-joining method [30].

Genomic DNA extracted from ‘Xushu55-2’, grown in vitro, was used to amplify the genomic sequence and the promoter of *IbWRKY2* [31]. The promoter region was cloned with Universal GenomeWalker 2.0 Kit (TaKaRa, Dalian, China) and the specific primers *IbWRKY2*-PROMOTER-1/2/3 (Table S1). The *cis*-acting regulatory elements in the promoter region of *IbWRKY2* were screened with PlantCARE (http://bioinformatics.psb.ugent.be/webtools/plantcare/html/).

### 2.3. Expression Analysis of IbWRKY2 in Sweetpotato

The transcript levels of *IbWRKY2* in the leaves, stems, hair roots, fibrous roots, and storage roots were measured with untreated Xushu55-2 plants. To further determine the transcript levels of *IbWRKY2* under different kinds of stresses, the roots of 4-week-old plants of Xushu55-2, grown in vitro, were soaked in Hoagland solution with 30% PEG6000, 200 mM NaCl, or 100 μM ABA and then sampled 0, 1, 3, 6, 12, and 24 h after treatment. The gene transcript levels were determined using quantitative real-time PCR (qPCR) with the primers *IbWRKY2*-qPCR-F/R displayed in Table S1, and *Ibactin* (AY905538) was employed as the internal control, as described by Liu et al. in 2014 (Table S1) [32]. The comparative *C*_T_ method was used to calculate the relative expression level [33]. Three technical replications and three biological replications for each sample were used to analyze the expression of *IbWRKY2*.

### 2.4. Subcellular Localization of IbWRKY2

The coding region of *IbWRKY2* was cloned and inserted into the *Pac*I/*Asc*I-digested pMDC83 expression vector containing the green fluorescent protein (*GFP*) gene (using the primers *IbWRKY2*-Loc-F/R) under the control of the CaMV35S promoter and nopaline synthase (NOS) terminator to generate the fusion protein IbWRKY2-GFP (Table S1). Both the fusion vector and the control vector were transformed into living onion epidermal cells by particle bombardment with a GeneGun (Biorad Helios^TM^, Hercules, California, USA) according to the instruction manual [31]. The onion cells were monitored using confocal microscopy at 488 nm 24–36 h after infiltration (Olympus, Tokyo, Japan).

### 2.5. Transactivation Assay of IbWRKY2 in Yeast

The full-length *IbWRKY2* coding sequence (CDS) was amplified by PCR using a pair of gene-specific primers (*IbWRKY2*-T7-F/R) and inserted into the *Nde*I/*Sal*I-digested pGBKT7 vector to produce the fusion construct pGBKT7-*IbWRKY2* (Table S1). The empty pGBKT7 vector was used as a negative control, and pGAL4 was used as a positive control. The fusion plasmid, negative control, and positive control were separately transformed into the yeast strain AH109. The transformed yeast was streaked on SD/−Trp and SD/−Trp/−His/X-α-Gal plates to observe yeast growth at 30 °C for 3–5 days.

### 2.6. Vector Construction and Arabidopsis Transformation

The full-length CDS of *IbWRKY2* was amplified by PCR using a pair of gene-specific primers (*IbWRKY2*-OE-F/R) and ligated to the *Sac*I/*Sal*I-digested pCAMBIA1300 vector (Table S1). The recombinant vectors were transferred into *Arabidopsis* through the *Agrobacterium tumefaciens* strain GV3101 using the floral dipping method [34]. The first generation (T_0_) seeds of transgenic *Arabidopsis* were germinated on 1/2 MS medium with 50 mg L^−1^ hygromycin for screening. Transgenic *Arabidopsis* plantlets were identified by PCR amplification and expression was confirmed using qPCR. The third-generation T_3_ homozygous lines were collected for all further experiments.

### 2.7. Drought and Salt Stress Treatment of Transgenic *Arabidopsis*

*IbWRKY2*-overexpressing *Arabidopsis* and WT seedlings were cultivated on 1/2 MS medium with 300 mM mannitol or 125 mM NaCl at 22 °C under 16 h of daylight. After 15 days, the primary root length and fresh weight were investigated. Meanwhile, transgenic *Arabidopsis* and WT seedlings were sown in pots and regularly watered for 2 weeks. The plants were subsequently irrigated with 100 mL of 300 mM NaCl solution for 2 weeks or subjected to withholding of watering for 2 weeks followed by 2 days of recommenced watering.

### 2.8. Measurement of Phytohormones and Stress-Related Components Contents

The ABA content was quantified by indirect enzyme-linked immunosorbent assay (ELISA) as described by Yang et al. [35]. The contents of proline and malondialdehyde (MDA) and the activity of superoxide dismutase (SOD) were measured using specific assay kits (Comin Biotechnology Co., Ltd., Suzhou, China). The H_2_O_2_ content in leaves was determined by 3,3′-diaminobenzidine (DAB) staining [36].

### 2.9. Expression Analysis of Stress-Tolerance-Related Genes in *Arabidopsis*

*IbWRKY2*-overexpressing *Arabidopsis* and WT plantlets grown in pots with or without abiotic stress were collected to analyze the expression of stress-tolerance-related genes. Genes involved in ABA signaling pathways, proline biosynthesis, and the ROS-scavenging system were analyzed using qPCR protocols, as described above. Primers specific for *Atactin* were used as an internal control; the details of all used primers are listed in Table S1. Three technical replicates corresponding to three biological replicates for each sample were used to assay gene expression.

### 2.10. Yeast Two-Hybrid Assay

The full-length and truncated CDSs of *IbWRKY2* were amplified by PCR using pairs of gene-specific primers (*IbWRKY2*-T7-F/F1/R/R1) and were ligated to the *Nde*I/*Sal*I-digested pGBKT7 bait vector to produce a fusion construct (Table S1). The longest sequence without self-transcriptional activation activity in the bait plasmid and prey plasmid library was co-transformed into the yeast strain AH109. The full-length CDSs of *IbVQ4* and *AtVQ4* were amplified by PCR using pairs of gene-specific primers (*IbVQ4*-T7-F/R and *AtVQ4*-T7-F/R) (Table S1). The bait and prey plasmids were co-transformed into the yeast strain AH109. The transformed yeast was examined on SD/−Trp/−Leu and SD/−Trp/−His/−Leu/−Ade/X-α-Gal plates to test for protein–protein interactions at 30 °C for 3–5 days.

### 2.11. Bimolecular Fluorescence Complementation (BiFC) Assay

Bimolecular fluorescence complementation (BiFC) assays were performed as described by Hu and Yu in 2014 [37]. The full-length *IbWRKY2* CDS was inserted into pSPYNE to form an in-frame fusion with the N-terminal region of YFP; meanwhile, the *IbVQ4* and *AtVQ4* CDSs were introduced into pSPYCE to generate an in-frame fusion with the C-terminal region of YFP. The plasmids were introduced into the *A. tumefaciens* strain AH105 and then injected into the leaves of *Nicotiana benthamiana.* The sequences of the specific primers *IbWRKY2*-nYFP-F/R, *IbVQ4*-cYFP-F/R, and *AtVQ4*-cYFP-F/R for vector construction are shown in Table S1. Infected leaves were analyzed 24–48 h after injection and monitored using confocal microscopy (Olympus, Tokyo, Japan). The two empty vectors were used as negative controls.

### 2.12. Expression Analysis of IbVQ4 in Sweetpotato

The transcript levels of *IbVQ4* were analyzed using qPCR according to the method described above; the specific primers IbVQ4-qPCR-F/R are shown in Table S1. Four-week-old plants of Xushu55-2, grown in vitro, were treated in Hoagland solution with either 30% PEG6000 or 200 mM NaCl and were sampled 0, 1, 3, 6, 12, and 24 h after treatment. Three technical replications and three biological replications for each sample were used to analyze the *IbVQ4* expression.

### 2.13. Statistical Analysis

All experiments were repeated three times, and the data are presented as the mean value ± SE. Statistical analysis was performed in Microsoft Excel 2010 with Student’s *t*-test (two-tailed analysis) at *p* < 0.05 (*) and *p* < 0.01 (**).

## 3. Results

### 3.1. Cloning and Sequence Analysis of *IbWRKY2* and Its Promoter

**Figure 1:**
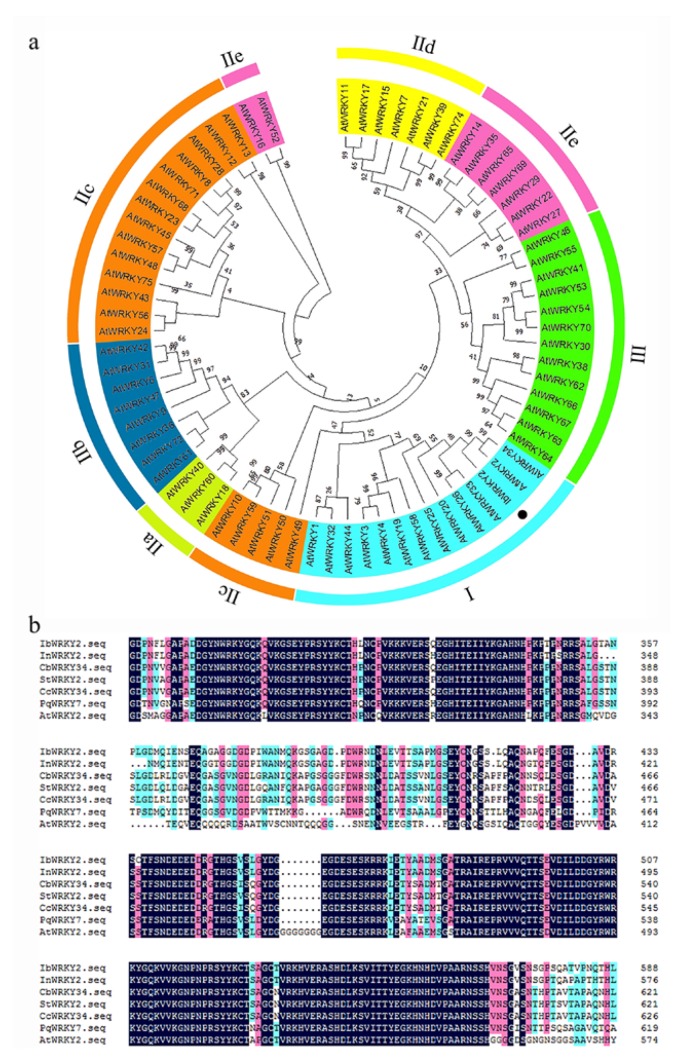
Phylogenetic relationship and conserved domain analysis of IbWRKY2. (**a**) Phylogenetic analysis of IbWRKY2 and 71 WRKY transcription factors from *Arabidopsis*. Roman numerals indicate different subfamilies of WRKY proteins and the black dot in the phylogenetic tree indicates IbWRKY2. (**b**) Amino acid sequence alignment of IbWRKY2 with homologs from NCBI. The conserved WRKY domains are marked by black lines.

**Figure 2:**
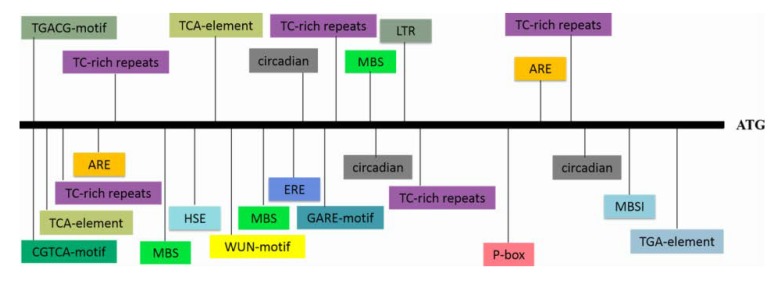
The types and locations of *cis*-acting elements present in the *IbWRKY2* promoter region. The promoter region, which is 2381 bp upstream of the initiation codon, was used for the analysis. Differently colored boxes represent different *cis*-acting elements. ATG indicates the start codon of *IbWRKY2*.

RNA-seq analysis in our previous study revealed an EST that was highly induced by PEG6000 stress; thus, this EST was selected for further characterization [29]. To obtain the full length of the differentially expressed EST, RACE-PCR was performed using the drought-tolerant sweetpotato line Xushu55-2. The ORF of this *WRKY* gene is 2133 bp and encodes a predicted protein of 710 aa with a molecular weight of 76.38 kDa and a predicted pI of 5.84. A phylogenetic tree was constructed with the amino acid sequences of this WRKY protein and 71 WRKY proteins of *Arabidopsis*. The result showed that this protein shared the highest identity with AtWRKY2 and AtWRKY34, and the amino acid sequence of this protein shared 51.98% and 43.00% similarity to AtWRKY2 and AtWRKY34, respectively; for this reason, it was designated as IbWRKY2 (Figure 1a). Multiple protein sequence alignments among IbWRKY2 and homeotic WRKYs of other plants showed that IbWRKY2 possessed two highly conserved WRKY domains, both composed of 58 aa, belonging to WRKY TF family group I (Figure 1b). A 2381 bp fragment corresponding to the promoter of *IbWRKY2* was isolated from Xushu55-2 genomic DNA using genome walking and was found to contain numerous types of *cis*-acting regulatory elements. Among these, several kinds of *cis*-acting regulatory elements involved in different biotic and abiotic stresses were identified, such as HSE, MBS, ERE, and GARE (Figure 2, Table S2). The presence of these stress-related *cis*-acting elements in the promoter regions indicates that the expression level of *IbWRKY2* might be influenced by different kinds of stresses (Figure 2, Table S2).

### 3.2. The Expression of *IbWRKY2* in Sweetpotato

**Figure 3:**
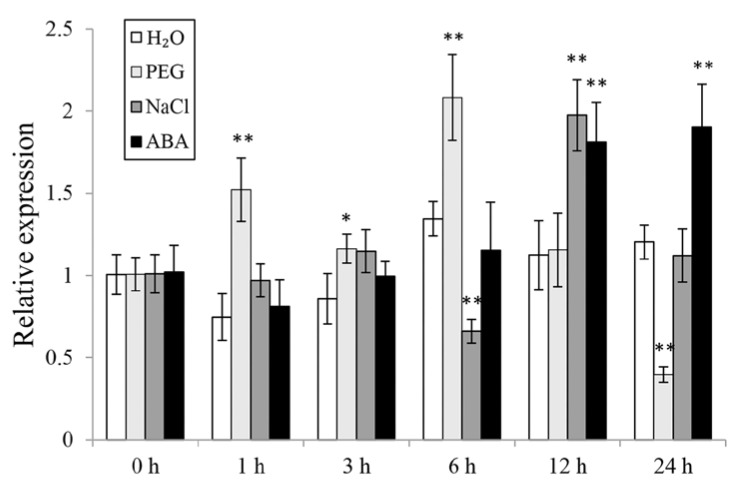
Expression level analysis of *IbWRKY2* in Xushu55-2 before and after H_2_O, 30% PEG6000, 200 mM NaCl, and 100 μM ABA treatments. Data are presented as means ± SE (n = 3). The time points 0, 1, 3, 6, 12, and 24 h indicate the time after the related treatment; * and ** indicate a significant difference at *p* < 0.05 and *p* < 0.01 compared with the related H_2_O control, respectively.

To investigate the potential working site of *IbWRKY2* in sweetpotato, we analyzed its expression level in different tissues of Xushu55-2, including the leaf, stem, hair root, fibrous root, and storage root. *IbWRKY2* showed a significantly higher expression level in leaves than in other tissues (Figure S1), indicating its critical function in sweetpotato leaves. To further analyze its potential function in response to abiotic stresses, the expression of *IbWRKY2* was checked using the whole plants of 4-week-old Xushu55-2, grown in vitro, that were treated with H_2_O, PEG6000, NaCl, or ABA for 0, 1, 3, 6, 12, and 24 h. After PEG6000 treatment, the expression level of *IbWRKY2* was strongly induced at 1 and 6 h (Figure 3). Moreover, based on available RNA-seq data, we found that the expression of *IbWRKY2* was induced by 25% PEG in sweetpotato (SRA data: SRX4522044, SRX4522043), similar to our result. Under NaCl stress, the expression of this gene was up-regulated at only 12 h (Figure 3). After ABA treatment, the expression of *IbWRKY2* was induced at 12 and 24 h (Figure 3). These results indicate that *IbWRKY2* might be involved in drought and salt signal response pathways.

### 3.3. IbWRKY2 is a Nuclear Protein with Transactivation Activity in Yeast

**Figure 4:**
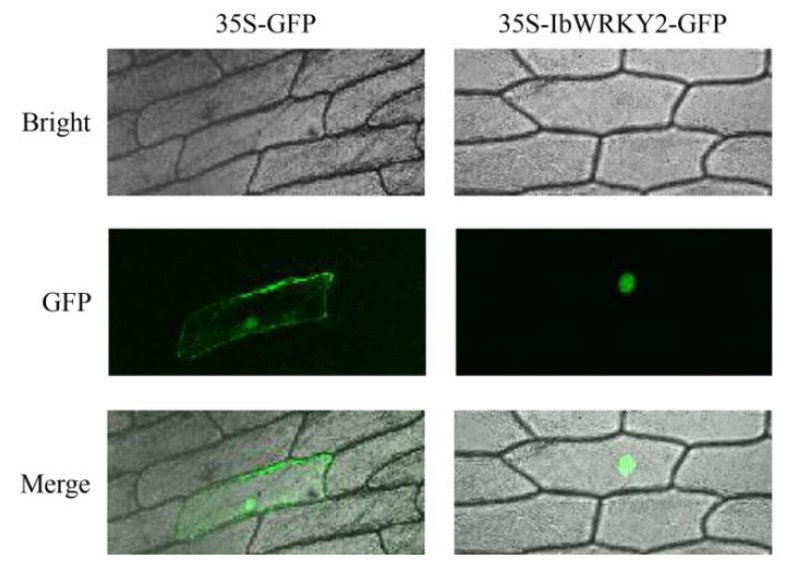
Subcellular localization of IbWRKY2 in onion epidermal cells. Confocal scanning microscopic images show that the IbWRKY2-GFP fusion protein localized to the nuclei (in the right column) vs. GFP as the control (in the left column).

**Figure 5:**
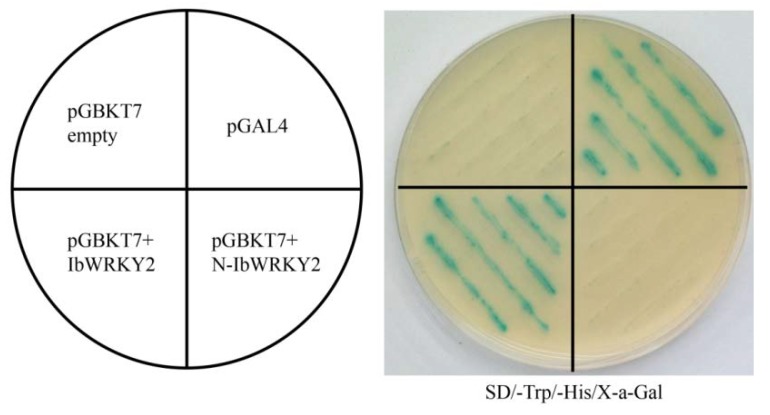
Transactivation activity assay of full-length and truncated IbWRKY2 in yeast. pGBK7+IbWRKY2, representing the full-length IbWRKY2, and pGBK7+N-IbWRKY2, representing IbWRKY2 with 246 aa removed from the N-terminal region, were transformed into the yeast strain AH109 and examined on SD/−Trp/−His selection medium with X-α-Gal. The pGBKT7 empty vector and pGAL4 were used as negative and positive controls, respectively.

**Figure 6:**
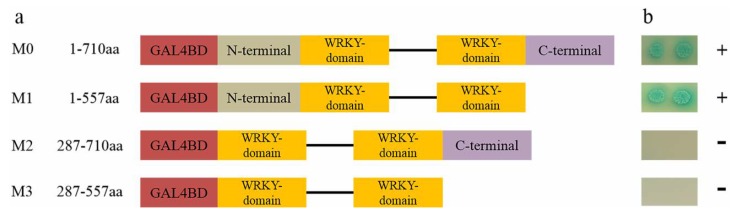
Diagram illustrating the region of IbWRKY2 involved in self-transcriptional activation activity and assay. (**a**) Schematic diagrams represent the IbWRKY2 fragments encoding different parts of IbWRKY2 that were cloned into the pGBKT7 vector. M1 is a C-terminal deletion mutant, M2 is an N-terminal deletion mutant, and M3 has both the C- and N-terminal regions deleted. The numbers denote IbWRKY2 amino acid positions. (**b**) Transactivation assay of IbWRKY2 and its deletion mutants. The transactivation activity is shown on the right (plus sign is positive and minus sign is negative).

To study the subcellular localization of IbWRKY2, the CDS of *IbWRKY2* was fused with GFP and transiently expressed in onion epidermal cells using the gene gun method. Confocal microscopic analysis showed that the IbWRKY2-GFP fusion protein specifically localized to nuclei, and the GFP fluorescence of the control was distributed throughout the whole cell (Figure 4). This result indicates that IbWRKY2 is a nuclear protein. To investigate the transactivation activity of IbWRKY2, the pGBKT7-*IbWRKY2* fusion construct, the pGBKT7 empty vector (negative control) and pGAL4 (positive control) were also separately transformed into the yeast strain AH109. Yeast cells containing any of the three vectors grew well on SD/−Trp medium; meanwhile, yeast cells containing pGBKT7-*IbWRKY2* and the positive control grew well on SD/−Trp/−His/X-α-Gal medium exhibiting α-galactosidase activity, while the ones containing the negative control did not grow (Figure 5). The full-length and truncated IbWRKY2 were tested for self-transcriptional activation activity (Figure 6a). The full-length IbWRKY2 and the truncated protein without the C-terminal region possessed self-transcriptional activation activity. After deleting 286 aa from the N-terminal region of this protein, the truncated IbWRKY2 lost its self-transcriptional activation activity (Figure 6b). These results indicate that IbWRKY2 acts as a transcription activator and that the self-transcriptional activation domain is located in the N-terminal region.

### 3.4. Overexpression of *IbWRKY2* Enhanced Drought and Salt Tolerance in Transgenic *Arabidopsis*

To further evaluate the function of *IbWRKY2*, it was transformed into *Arabidopsis* to generate overexpression lines. A total of 9 transgenic lines were obtained and confirmed by PCR. We named these overexpression lines OE1–OE9 (Figure S2). The homozygous T_2_ plants that showed no segregation on the selective medium were selected to produce T_3_ homozygous lines for further experiments. Three positive transgenic lines OE1, OE2, and OE4 were found to show much higher expression levels of *IbWRKY2* than wild type (WT) and other transgenic lines detected by qPCR, so these three lines were selected for further research (Figure S3).

**Figure 7:**
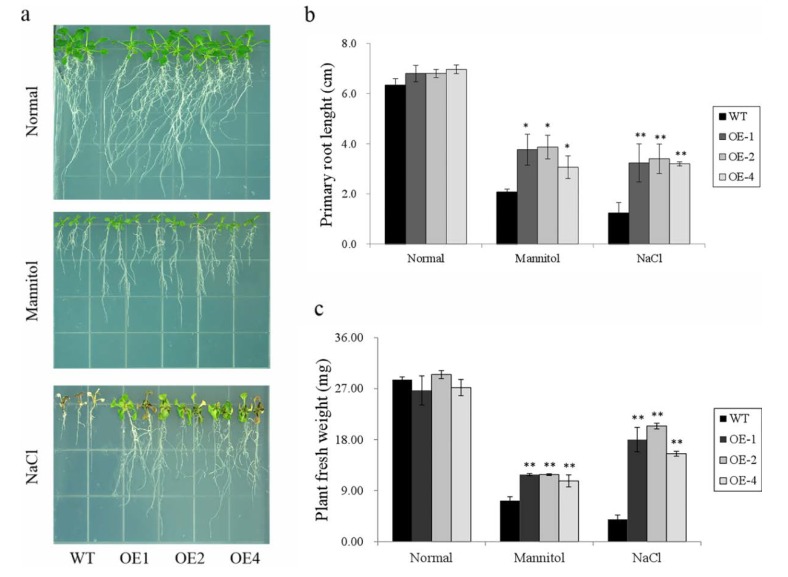
Responses of the transgenic *Arabidopsis* and WT seedlings cultured on MS medium with no stress, 300 mM mannitol, or 125 mM NaCl for 15 days. (**a**) Phenotype of WT and transgenic lines grown under different stresses; (**b**) primary root length of the WT and transgenic lines shown in (**a**); and (**c**) fresh weight of the WT and transgenic lines shown in (**a**). Data are presented as means ± SE (n = 3); * indicates a significant difference at *p* < 0.05, and ** means a significant difference at *p* < 0.01.

Five-day-old transgenic plants and WT seedlings were used for stress treatment tests. The plants were grown on 1/2 MS medium with 300 mM mannitol or 125 mM NaCl for 15 days. The primary root length and fresh weight were measured as indicators of the stress tolerance of plants. No significant difference in growth was observed among the three overexpression lines and the WT when they were cultured on normal 1/2 MS medium (Figure 7a–c). After stress treatments, the primary root length and fresh weight decreased in both the WT and transgenic lines compared with the normal condition, but the degrees of reduction were different (Figure 7a). The primary root length of the overexpression lines was significantly longer than that of the WT, and the fresh weight of transgenic lines was also considerably greater than that of the WT under both mannitol and salt stress treatments (Figure 7b,c). Together, these results indicate that the transgenic lines had significantly better morphological growth (Figure 7a–c) corresponding to better stress tolerance.

**Figure 8:**
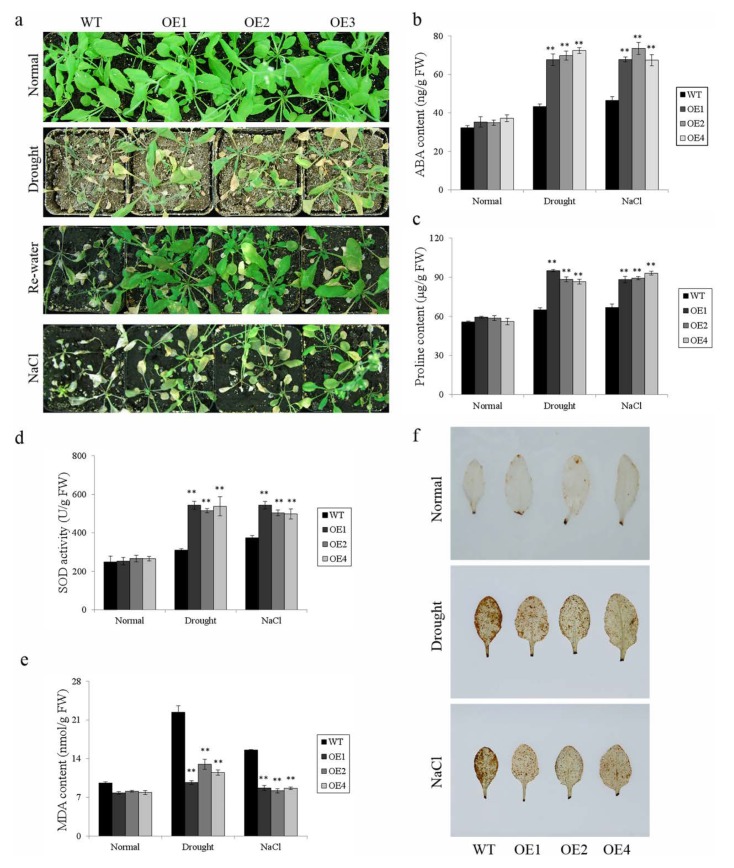
Responses of transgenic *Arabidopsis* and WT plants grown in pots under drought and salt stresses. (**a**) Phenotypes of transgenic lines and WT grown for 4 weeks under normal condition, 2 weeks under normal condition, and 2 weeks under 300 mM NaCl treatment or 2 weeks under normal conditions and 2 weeks under drought stress followed by 2 days of re-watering; (**b**) ABA content in transgenic lines and WT under the normal or stress condition shown in (**a**); (**c**) proline content in transgenic lines and WT under the normal or stress condition shown in (**a**); (**d**) SOD activity in transgenic lines and WT under the normal or stress condition shown in (**a**); (**e**) MDA content in transgenic lines and WT under the normal or stress condition shown in (**a**); and (**f**) H_2_O_2_ content indicated by DAB staining in transgenic lines and WT under the normal or stress condition. Data are presented as means ± SE (n = 3); ** indicate a significant difference at *p* < 0.01.

To further verify whether *IbWRKY2* contributes to the tolerance of drought and salt stresses, we performed pot experiments using the same transgenic lines and WT plants. After growing in pots for 2 weeks, the transgenic lines and WT were treated with 300 mM NaCl or drought stress. The transgenic lines and WT grew well and showed no significant difference under normal conditions (Figure 8a–f). Meanwhile, the WT plants exhibited increased sensitivity to abiotic stresses compared with the transgenic lines, as shown in Figure 8a. The content of ABA was considerably higher in the transgenic lines than in the WT (Figure 8b). The transgenic lines contained significantly lower MDA, higher proline, and higher SOD activity than the WT (Figure 8c–e). DAB staining indicated that the leaves of the transgenic plants accumulated less H_2_O_2_ than those of WT (Figure 8f). All these results indicate that overexpression of *IbWRKY2* enhances drought and salt tolerance in *Arabidopsis*.

### 3.5. Overexpression of *IbWRKY2* Activates the Expression of the Stress-Responsive Genes

**Figure 9:**
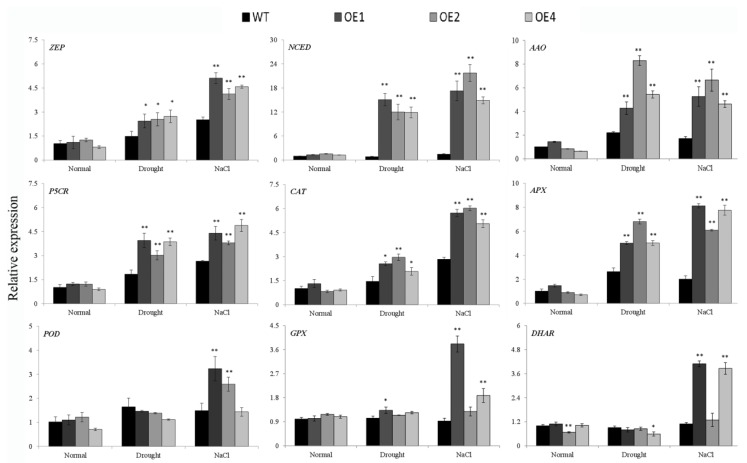
Transcript levels of drought- and salt-responsive genes in transgenic *Arabidopsis* and WT plants. The plants were grown under normal conditions for two weeks and then used for normal, NaCl, and drought stress treatments. After one week of treatment, the leaves of transgenic lines and WT were sampled for analysis. Data are presented as means ± SE (n = 3); * and ** indicate a significant difference at *p* < 0.05 and *p* < 0.01, respectively. The gene expression in WT under normal conditions was set to 1, and the expression in the other samples was adjusted accordingly.

To investigate the reason that *IbWRKY2* affected drought and salt resistance in transgenic plants, we analyzed the expression of several genes involved in different pathways. Under normal conditions, most of the gene expression levels showed no obvious differences between the WT and transgenic plants, except for *AtDHAR* in OE2 (Figure 9). After drought or salt stress, the expression levels of the ABA signal transduction pathway–related genes zeaxanthin epoxidase (ZEP), 9-*cis*-epoxycarotenoid dioxygenase (NCED), and ABA-aldehydeoxidase (AAO) and the proline-biosynthetic-pathway gene pyrroline-5-carboxylate reductase (P5CR) significantly increased in transgenic lines compared with the WT (Figure 9). The ROS-scavenging genes that encode catalase (CAT) and ascorbate peroxidase (APX) also showed obvious up-regulation in transgenic lines after drought or salt treatment (Figure 9). Meanwhile, the ROS-scavenging genes peroxidase (POD), glutathione peroxidase (GPX), and dehydroascorbate reductase (DHAR) were up-regulated in only some transgenic lines after salt or drought treatment (Figure 9). These results indicate that overexpression of *IbWRKY2* improves stress tolerance by activating the expression of genes involved in the ABA signal transduction pathway, proline biosynthetic pathway, and the ROS-scavenging system under drought and salt treatments.

### 3.6. IbWRKY2 Interacts with VQ4

**Figure 10:**
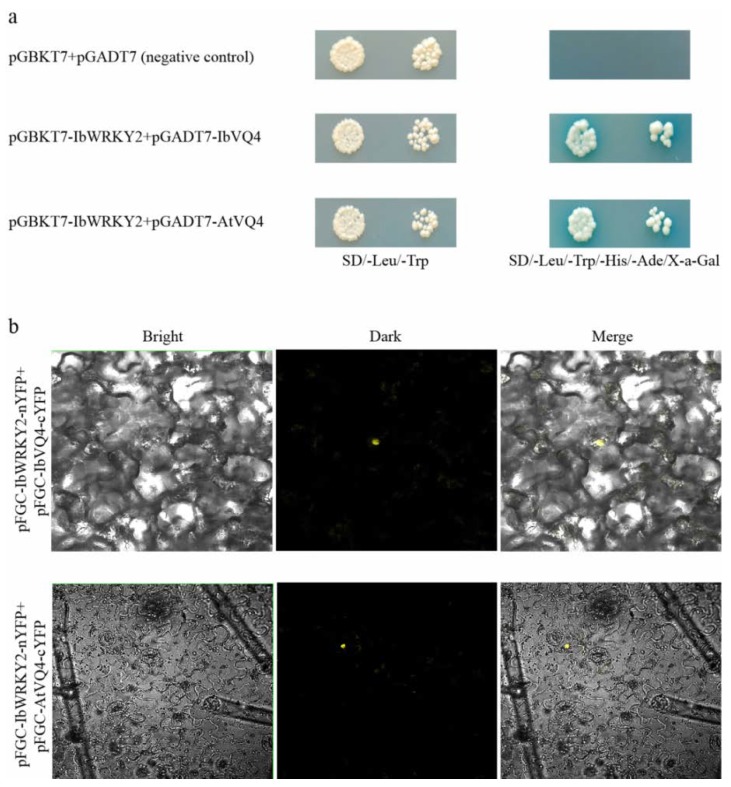
Protein interaction assays between IbWRKY2 and VQ4. (**a**) Yeast two-hybrid assay showing the interaction between IbWRKY2 and VQ4 proteins from sweetpotato and *Arabidopsis*; pGBKT7 and pGADT7 plasmids served as negative controls. (**b**) BiFC visualization of the interaction between IbWRKY2 and VQ4 proteins from sweetpotato and *Arabidopsis* in tobacco leaves.

To investigate the *IbWRKY2* mechanisms involved in stress regulation, we screened the yeast two-hybrid library to find IbWRKY2 interaction proteins. Several candidate interaction proteins were obtained by the yeast two-hybrid assay between truncated IbWRKY2 and prey sweetpotato cDNA library. The novel protein IbVQ4, which contains a VQ motif, was selected and cloned for further analysis. The transformed yeast cells that contained both pGBKT7-IbWRKY2 and pGADT7-IbVQ4 showed good growth and exhibited α-galactosidase activity on SD/−Trp/−His/−Leu/−Ade/X-α-Gal medium, which demonstrated the interaction of these two proteins in yeast (Figure 10a). In the BiFC assay, YFP fluorescence could be detected in the cells of *N. benthamiana* leaves after they were injected with *A. tumefaciens* AH105 containing pSPYNE-IbWRKY2-nYFP and pSPYCE-IbVQ4-cYFP, which further indicated an interaction between these two proteins (Figure 10b). To study the functional mechanism of *IbWRKY2* in transgenic *Arabidopsis*, an *IbVQ4* homologous gene, *AtVQ4*, was also cloned into the pGADT7 and pSPYCE vectors. Both the yeast two-hybrid and BiFC assays confirmed that IbWRKY2 could interact with the AtVQ4 protein (Figure 10a, b). Further qPCR analysis of *IbVQ4* before and after stress treatments showed that the expression level of this gene was also induced by drought and salt stresses, which indicates that IbVQ4 might be related to stress resistance in sweetpotato (Figure S4).

## 4. Discussion

### 4.1. Overexpression of *IbWRKY2* Enhances Drought and Salt Stress Tolerance

TFs, as switches of gene transcription, have been reported to be necessary for many biological processes [38]. WRKY TF members are named according to the highly conserved WRKY domain and are divided into three groups depending on the number of WRKY domains and the type of zinc finger [15]. WRKY, representing one of the largest TF families specific to plants, has been reported to be involved in growth and development, defense regulation, and the stress response [13]. The first gene in this family was cloned from sweetpotato, after which numerous members were cloned in *Arabidopsis*, rice, soybean, tomato, and maize [39,40,41,42,43,44]. In recent years, several TFs have been confirmed to be involved in the regulation of drought or salt tolerance in different kinds of plants [17,45,46,47]. To date, several TFs have been confirmed to be involved in the regulation of drought or salt tolerance, but few WRKY family members have been studied in sweetpotato [48,49,50].

Here, we isolated a novel WRKY TF member according to the EST obtained from the transcriptome data of our previous study [29]. This protein contains two conserved WRKY domains belonging to group I and has a close relationship with AtWRKY2; thus, the novel protein was named IbWRKY2 (Figure 1). IbWRKY2 is located in the nucleus and possesses a transcriptional activation domain in its N-terminal region (Figure 4 and Figure 5). In further studies, *IbWRKY2* was strongly induced by PEG, NaCl, and ABA treatments, and its overexpression enhanced drought and salt tolerance in transgenic *Arabidopsis* (Figure 3, Figure 7 and Figure 8). In previous studies, *AtWRKY2* has been demonstrated to be necessary for pollen development, *CsWRKY2* has been shown to be induced by drought and cold stress, and *TaWRKY2* may enhance salt and drought tolerance. However, no further evidence has supported a relationship between *IbWRKY2* and abiotic stress tolerance [4,23,24].

### 4.2. Overexpression of *IbWRKY2* Activated the ABA Signaling Pathway

ABA plays a significant role in the response to abiotic stresses; it promotes the closure of stomata in guard cells to retain water and also regulates the expression of stress-tolerance-related genes [51,52]. A growing body of evidence has indicated that many WRKY TFs regulate stress tolerance through the ABA signal transduction pathway [5,47]. WRKY TFs can act as both positive and negative regulators of ABA-induced stomatal closure [53]. At the same time, they also appear to act both upstream and downstream in the ABA signal transduction pathway [54,55]. In the present study, we demonstrated that *IbWRKY2* expression was significantly up-regulated by PEG, NaCl, and ABA treatments in sweetpotato, and in *IbWRKY2* transgenic lines, the ABA content was higher than that in WT under drought and salt stress (Figure 3 and Figure 8b). Meanwhile, several genes related to ABA biosynthesis and the ABA signal transduction pathway were more dramatically up-regulated in transgenic lines than in WT (Figure 9). These results suggest that *IbWRKY2* might act as a positive regulator in drought and salt tolerance in an ABA-dependent manner.

### 4.3. Overexpression of *IbWRKY2* Results in Changes to the ROS-Scavenging System

ROS is important in the process of signal transduction that mediates tolerance to different stresses, but its excessive accumulation causes damage in plants [56]. Low level of ROS can activate the stress response while high levels of ROS will attack DNA, proteins, and carbohydrates [57]. Therefore, a certain level of ROS in plant cells is harmless and, in fact, critical for modulating the balance between ROS-producing and ROS-scavenging systems [58]. Increased proline content was shown to enhance salt and drought tolerance through the up-regulation of ROS-scavenging genes in transgenic sweetpotato [59]. In the present study, greater proline accumulation was found in *IbWRKY2*-overexpressing *Arabidopsis* after stress treatments (Figure 8c). As a result, ROS-scavenging genes were up-regulated in the transgenic plants under drought and salt treatments (Figure 9). The stimulated ROS-scavenging system led to lower MDA and H_2_O_2_ contents, which resulted in enhanced drought and salt tolerance (Figure 8e,f).

### 4.4. IbWRKY2 Improved Abiotic Stress Tolerance by Interacting with VQ4

In the last several years, it has been demonstrated that WRKY TFs physically interact with specific types of proteins. Different patterns have been discovered in the interactions between WRKY members and proteins, such as the WRKY-R model, WRKY-14-3-3 model, WRKY-WRKY model, WRKY-MAPK model, WRKY-chromatin model, and WRKY-VQ model [19,60,61,62,63,64]. VQ proteins are plant-specific proteins with a highly conserved VQ motif possessing the core sequence FxxhVQxhTG [65]. VQ proteins can specifically interact with the C-terminal WRKY domains of group I proteins and sole WRKY domains of group IIc proteins [26]. In *Arabidopsis*, several WRKYs that interact with VQ proteins have been identified to be involved in stress responses. For example, AtWRKY8 interacts with AtVQ10 to modulate defense against *Botrytis cinerea*, AtWRKY8 interacts with AtVQ9 to participate in salinity stress tolerance, and AtVQ16 and AtVQ23 can interact with WRKY33 to positively regulate plant defense [19,66,67]. Many VQ motif-containing proteins are considered to be involved in stress responses. For example, in poplar, the expression of numerous *VQ* genes, such as *PtVQ1*, *PtVQ4*, and *PtVQ11*, were induced by drought and salt stresses [68]. Nevertheless, no research on the interaction of VQ proteins in sweetpotato has been reported till now. In our present study, a protein containing a VQ motif was found to interact with IbWRKY2 through yeast two-hybrid. The protein was named IbVQ4 according to its close relationship with AtVQ4. Both the yeast two-hybrid and BiFC assays confirmed the interaction between IbWRKY2 and IbVQ4, which might reveal the functional mechanism of *IbWRKY2* (Figure 10a,b). Further research on the relationship between IbWRKY2 and AtVQ4, the homologous protein of IbVQ4, also supported an interaction between these two proteins (Figure 10a,b). In addition, our results showed that the expression level of *IbVQ4* was induced by PEG and NaCl treatments, which indicates that this gene is probably involved in drought and salt tolerance in sweetpotato (Figure S4). The discovery of this interaction protein offers insights into the potential molecular mechanism by which *IbWRKY2* improves drought and salt tolerance in sweetpotato.

## 5. Conclusions

A sweetpotato group I WRKY gene, *IbWRKY2*, was isolated and characterized for the first time, and its characteristics were investigated in this study. IbWRKY2 was found to be nuclear-localized, consistent with functioning as a transcription factor and it was implicated in transcriptional activation. The self-transcriptional activation domain was located in the N-terminal region. *IbWRKY2* was expressed with high abundance in leaves and induced by PEG6000, NaCl, and ABA stresses. Overexpression of *IbWRKY2* in *Arabidopsis* increased the plant’s tolerance to drought and salt stress by interacting with VQ4 proteins and regulating the expression of genes related to the ABA signaling pathway, proline biosynthesis, and the ROS-scavenging system. The results of this study indicate the functioning of a novel gene in enhancing the tolerance to abiotic stress in sweetpotato. The precise functional mechanism of *IbWRKY2* is worthy of further research.

## Supplementary Materials

The following are available online at https://www.mdpi.com/2218-273X/10/4/506/s1, Figure S1: Expression level analysis of *IbWRKY2* in different tissues of Xushu55-2. Data are presented as means ± SE (n = 3). HR, hair root; FR, fibrous root; SR, storage root. Figure S2: PCR identification of *IbWRKY2*-overexpressing *Arabidopsis* plants. M, marker; P, plasmid pCAMBIA3301-*IbWRKY2* as a positive control; WT, negative control; OE1-OE9, transgenic plants. Figure S3: Relative expression level of *IbWRKY2* in WT and transgenic lines. The *Arabidopsis* *Atactin* was used as an internal control. Data are presented as means ± SE (n = 3). Figure S4: Expression level analysis of *IbVQ4* in Xushu55-2 before and after treatment with 30% PEG6000 and 200 mM NaCl. Data are presented as means ± SE (n = 3). Table S1: Primers used in this study. Table S2: The *cis*-acting motifs detected in the promoter region of the *IbWRKY2* gene.
