# Supplementary material for: A Novel Sweetpotato WRKY Transcription Factor, IbWRKY2, Positively Regulates Drought and Salt Tolerance in Transgenic Arabidopsis
Source: Biomolecules. 2020 Mar 27;10(4):506. doi: 10.3390/biom10040506 (PMC7226164; doi:10.3390/biom10040506)
Supplement: Supplementary file 1 [file biomolecules-10-00506-s001.zip › WRKY2-Supplementary material.pdf]

Table S1. Primers used in this study

Primer name	Primer scequence (5′-3′)
Ibactin-F	AGCAGCATGAAGATTAAGGTTGTAGCAC
Ibactin-R	TGGAAAATTAGAAGCACTTCCTGTGAAC
IbWRKY2-qPCR-F	ATGCAGTGGATCGGTCTTGTAC
IbWRKY2-qPCR-R	CCCTAATGGCTCTGGTAGCG
IbWRKY2-5′RACE-OUTER	AGACGGCTGTGCCAGTGAAT
IbWRKY2-5′RACE-INNER	CCCATCACTATTTCTCGACGAAG
IbWRKY2-3′RACE-OUTER	GCAGCCCGCAATAGTAGTCA
IbWRKY2-3′RACE-INNER	CCTCTTTCACTCGGTTCATTTG
IbWRKY2 -F	ATGGGTGGATTTGATGACCATG
IbWRKY2 -R	TTACATTTGAGGTCCAAGAGGCA
IbWRKY2-PROMOTER-1	GACCAGGAGGAATCGTCAAGTAAG
IbWRKY2-PROMOTER-2	CACCACCTTTCCCATCACTATTTC
IbWRKY2-PROMOTER-3	CCAGGATTGATGAGAAAAACGC
IbWRKY2-Loc-F	CCTTAATTAAATGGGTGGATTTGATGACCATG
IbWRKY2-Loc-R	AGGCGCGCCACATTTGAGGTCCAAGAGGCA
IbWRKY2-T7-F	GGAATTCCATATGATGGGTGGATTTGATGACCATG
IbWRKY2-T7-R	ACGCGTCGACTTACATTTGAGGTCCAAGAGGCA
IbWRKY2-OE-F	CGAGCTCATGGGTGGATTTGATGACCATG
IbWRKY2-OE-R	ACGCGTCGACCATTTGAGGTCCAAGAGGCAAC
IbWRKY2-T7-F1	CCTTAATTAAGATGATGGTTATAATTGGAGG
IbWRKY2-T7- R1	TTGGCGCGCCGTGGTTGTGCTTCCCTTCAT
IbVQ4-T7-F	GGAATTCCATATGATGACTTCAATGGCTCATCATCAAG
IbVQ4-T7-R	CGGGATCCTTAGTCGGCTCCGGGCG
AtVQ4-T7-F	GGAATTCCATATGATGGAGATTTCAACAAACCCACC
AtVQ4-T7-R	CGGGATCCCTACATCTCCGGCGATAATCTC
IbWRKY2-nYFP-F	AGGCGCGCCATGGGTGGATTTGATGACCATG
IbWRKY2-nYFP-R	CCGCTCGAGCATTTGAGGTCCAAGAGGCA
IbVQ4-cYFP-F	AGGCGCGCCATGACTTCAATGGCTCATCATCAAG

IbVQ4-cYFP-R	GGGGTACCGTCGGCTCCGGGCG
AtVQ4-cYFP-F	AGGCGCGCCATGGAGATTTCAACAAACCCACC
AtVQ4-cYFP-R	GGGGTACCCATCTCCGGCGATAATCTC
Atactin-F	GCACCCTGTTCTTCTTACCGA
Atactin-R	AGTAAGGTCACGTCCAGCAAGG
AtZEP-F	CGGAGCTTTCTTCTTGATGG
AtZEP-R	TCGATTTCGGAGTTTTCCTG
AtNCED-F	CGCCGGTTTAGTTTATTTCAATGGT
AtNCED-R	AATCGTACCGACCCGAAGTTTCTAA
AtAAO-F	CAACAGCCATGTTGATACCG
AtAAO-R	TCTTTGACCTGCACATCGAG
AtP5CR-F	AGTTTAGCTTCACAGACCGTTC
AtP5CR-R	GCTCTGTGAGAGCTCGCGGCTTC
AtCAT-F	GCAACTACCCCGAGTGGAAA
AtCAT-R	TGTTCAGAACCAAGCGACCA
AtAPX-F	CTCTGGGACGATGCCACAAG
AtAPX-R	CTCGACCAAAGGACGGAAAA
AtPOD-F	TCCGGGAGCCACACCATTGG
AtPOD-R	TGGTCGGAATTCAACAG
AtGPX-F	ATGGCGACGAAGGAACCAG
AtGPX-R	ATCGCCGAAGATTCCCCATTT
AtDHAR-F	ATGGTCCTTTTATCGCCGGG
AtDHAR-R	GCCCATCCAGAGATCACACA
IbVQ4-qPCR-F	TTCTTGATTTCCCGTCGCTC
IbVQ4-qPCR-R	TCTTCTCCGCTATGGCTTTCTC

Table S2. *Cis*-acting motifs detected in the promoter region of the *IbWRKY2* gene

Position	Name	Sequence	Predicted function
-2148 -398	ARE	TGGTTT	anaerobic induction
-2361	CGTCA-motif	CGTCA	MeJA response
-1385	ERF	ATTTCAAA	ethylene response
-2361	TGACG-motif	CGTCA/TGACG	MeJAresponse
-2281 -1661	TCA-element	CCATCTTTTT	Salicylic acid response
-56	TGA-element	AACGAC	Auxin response
-1706	HSE	AGAAAATTCG	Heat stress response
-811	LTR	CCGAAA	Low temperature response
-902 -1403 -1813	MBS	C/TAACTG	Drought stress response
-2066 -2180 -959	TC-rich repeats	A/GTTTTCTC/TCA	Defense and stress response
-678	Skn-1_motif	GTCAT	Endosperm expression element
-1616	WUN-motif	AAATTTCCT	Wound response
-1037	GARE-motif	AAACAGA	Gibberellin response
-229	MBSI	TTTTTACGGTTA	Flavonoid regulation
-412	P-box	CCTTTTG	Gibberellin response

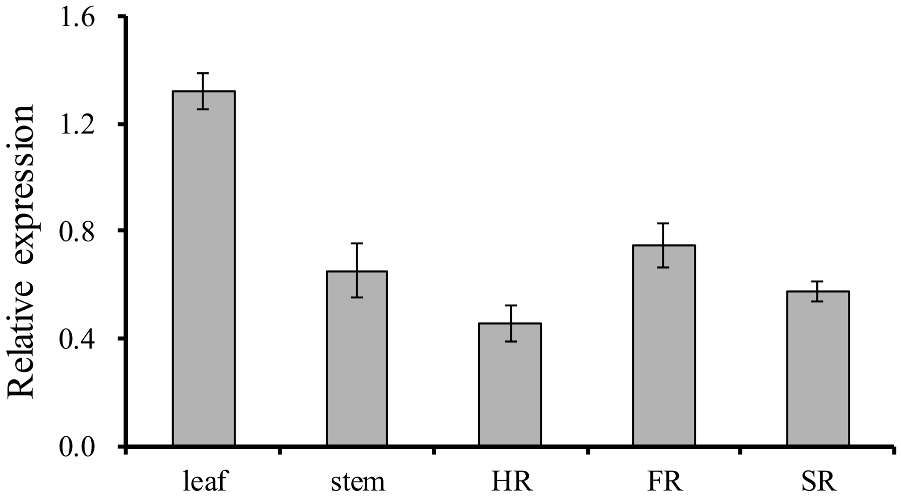

Figure S1. Expression level analysis of *IbWRKY2* in different tissues of Xushu55-2. Data are presented as means ± SE (n = 3). HR, Hair root; FR, Fibrous root; SR, Storage root.

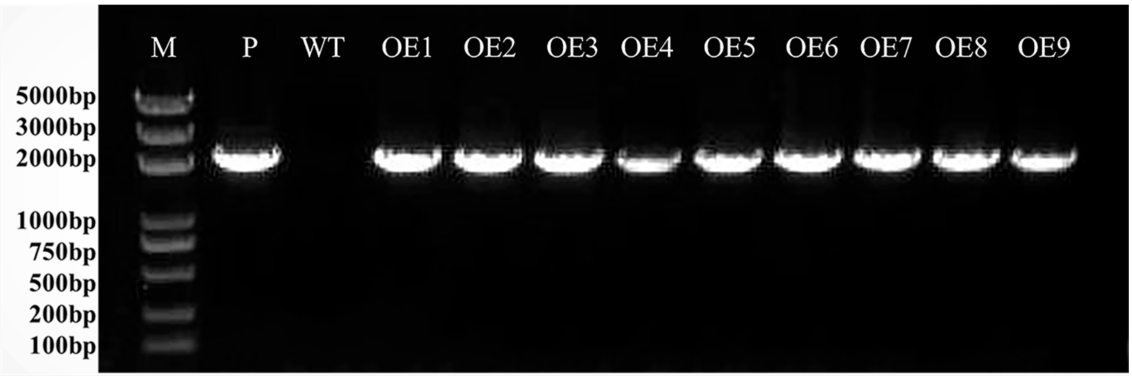

Figure S2. PCR identification of the *IbWRKY2*-overexpressing *Arabidopsis* plants. M, marker; P, plasmid pCAMBIA3301-*IbWRKY2* as positive control; WT, negative control; OE1-OE9, transgenic plants.

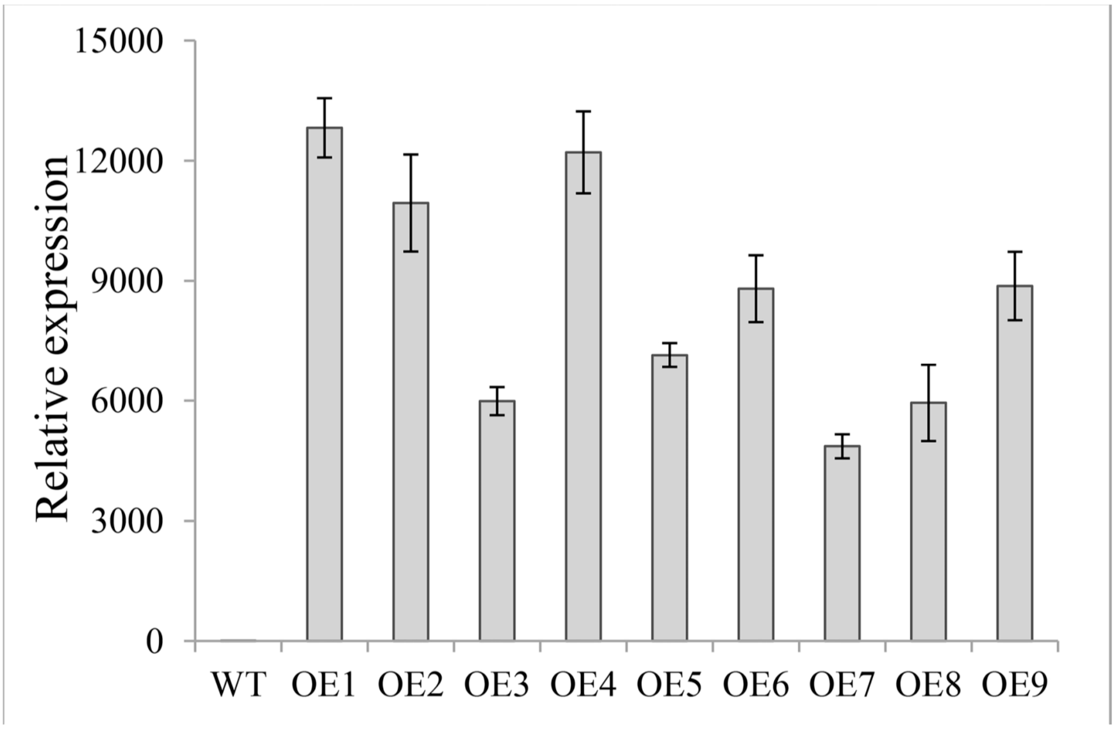

Figure S3. Relative expression level of *IbWRKY2* in WT and transgenic lines. The *Arabidopsis actin* gene was used as an internal control. Data are presented as means ± SE (n=3).

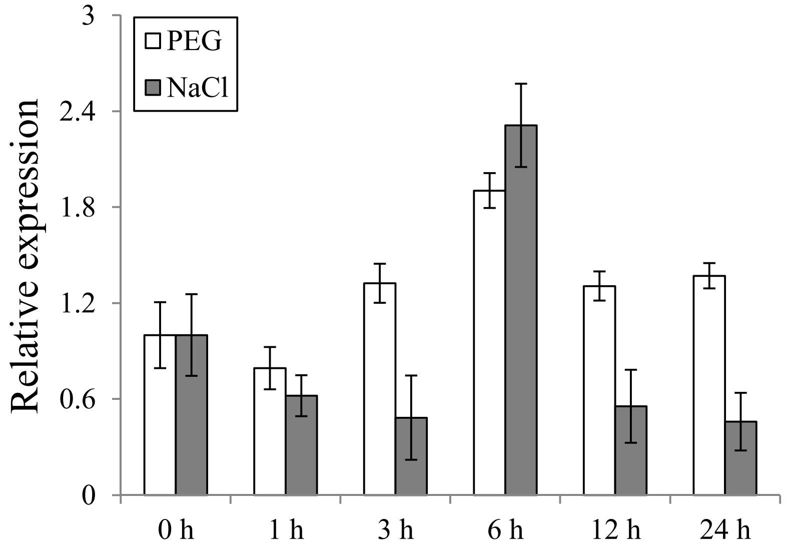

Figure S4. Expression level analysis of *IbVQ4* in Xushu55-2 before and after 30% PEG6000 and 200 mM NaCl. Data are presented as means ± SE (n = 3).
