# Supplementary figures and images for: A Novel Sweetpotato WRKY Transcription Factor, IbWRKY2, Positively Regulates Drought and Salt Tolerance in Transgenic Arabidopsis
Source: Biomolecules. 2020 Mar 27;10(4):506. doi: 10.3390/biom10040506 (PMC7226164; doi:10.3390/biom10040506)

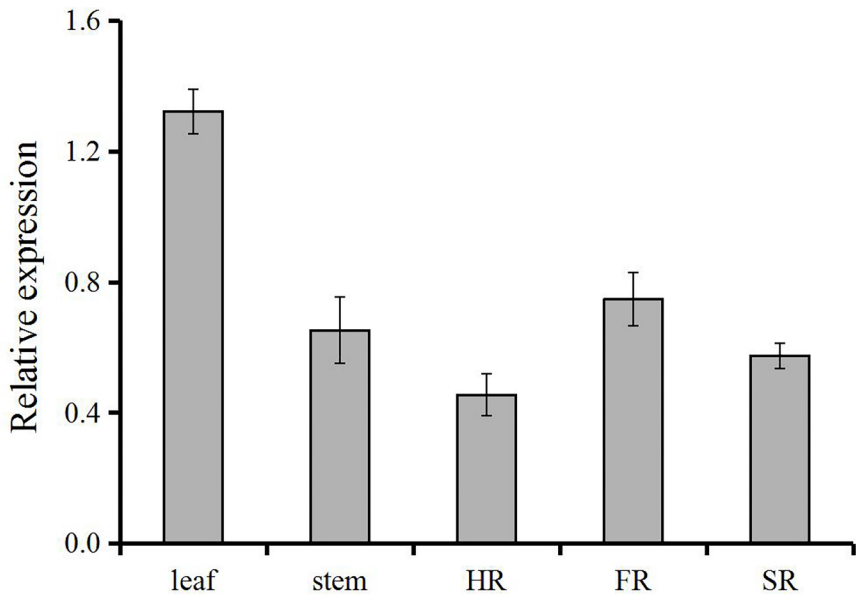

Supplement: Supplementary file 1 [file biomolecules-10-00506-s001.zip › figureS1.pdf]

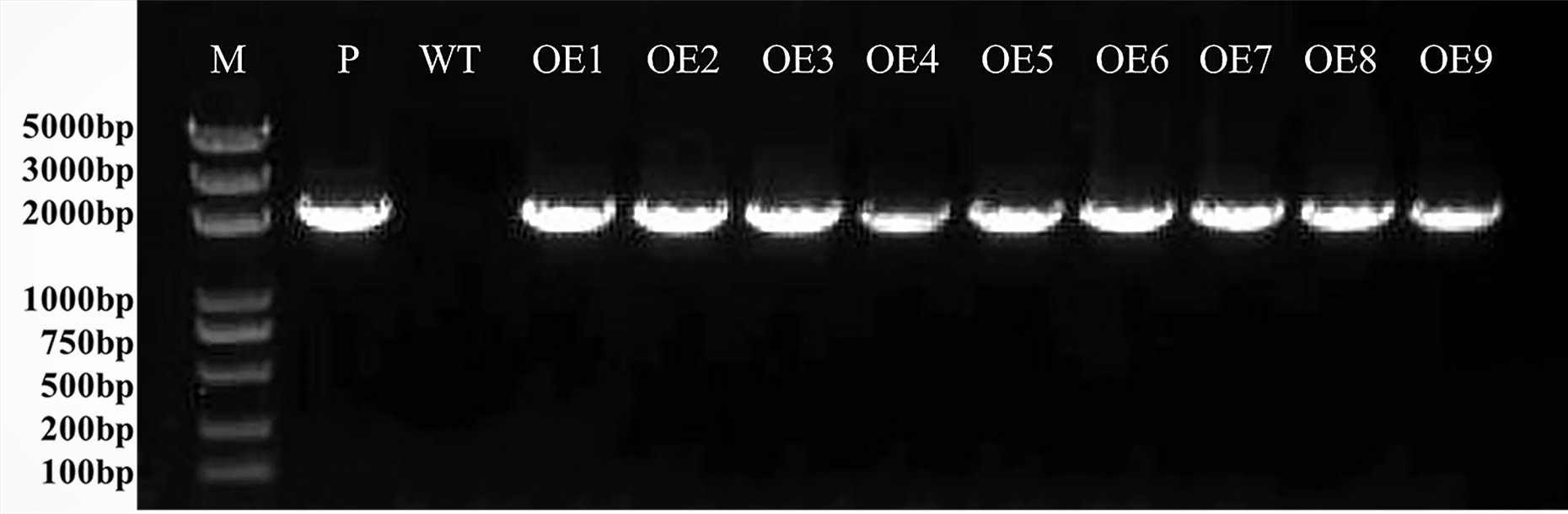

Supplement: Supplementary file 1 [file biomolecules-10-00506-s001.zip › figureS2.pdf]

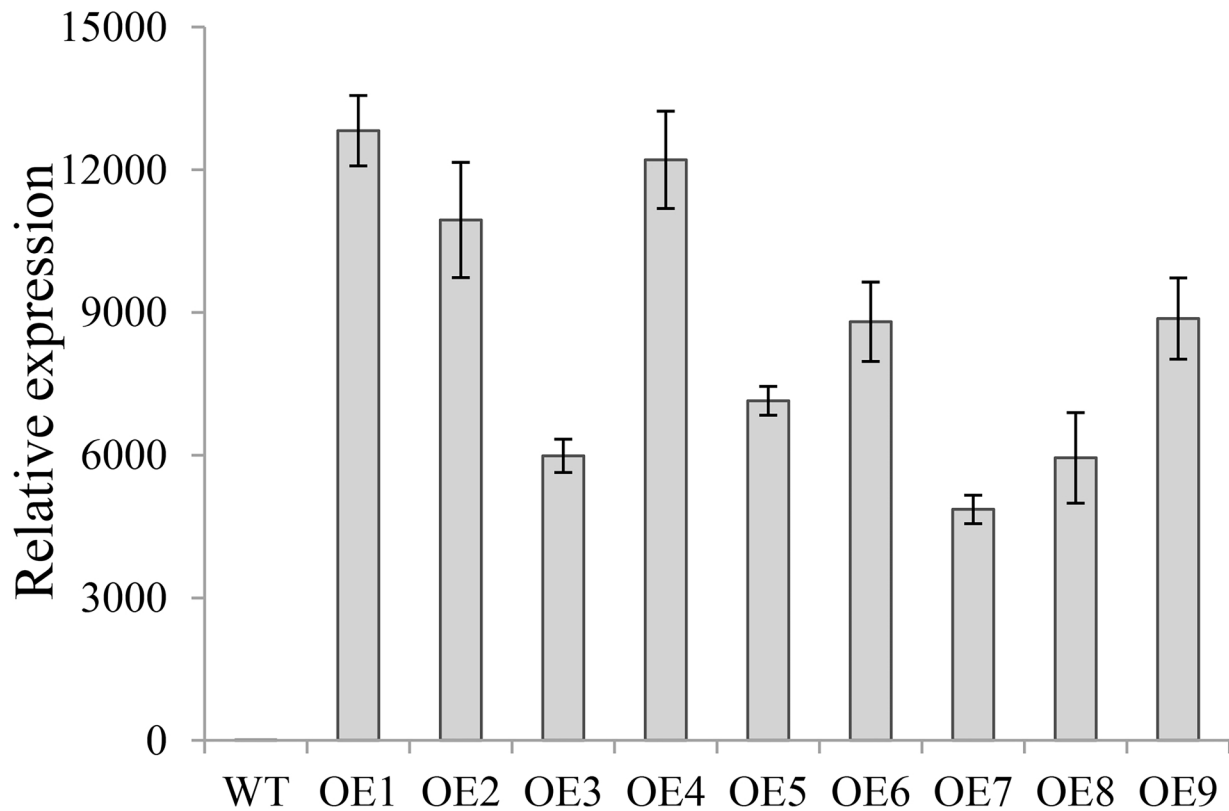

Supplement: Supplementary file 1 [file biomolecules-10-00506-s001.zip › figureS3.pdf]

Relative expression

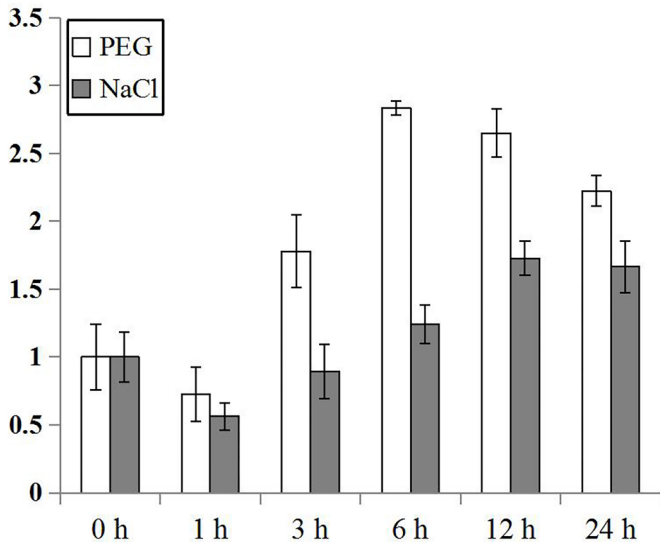

Supplement: Supplementary file 1 [file biomolecules-10-00506-s001.zip › figureS4.pdf]
